# Targeted Thrombospondin-1 Expression in Ocular Vascular Development and Neovascularization

**DOI:** 10.3389/fcell.2021.671989

**Published:** 2021-04-21

**Authors:** Christine M. Sorenson, Shoujian Wang, Soesiawati R. Darjatmoko, Zafer Gurel, Bo Liu, Nader Sheibani

**Affiliations:** ^1^Department of Pediatrics, University of Wisconsin School of Medicine and Public Health, Madison, WI, United States; ^2^McPherson Eye Research Institute, University of Wisconsin School of Medicine and Public Health, Madison, WI, United States; ^3^Department of Ophthalmology and Visual Sciences, University of Wisconsin School of Medicine and Public Health, Madison, WI, United States; ^4^Department of Human Oncology, University of Wisconsin School of Medicine and Public Health, Madison, WI, United States; ^5^Department of Surgery, University of Wisconsin School of Medicine and Public Health, Madison, WI, United States; ^6^Department of Cell and Regenerative Biology, University of Wisconsin School of Medicine and Public Health, Madison, WI, United States; ^7^Department of Biomedical Engineering, University of Wisconsin School of Medicine and Public Health, Madison, WI, United States

**Keywords:** retinal vasculature, choroidal vasculature, endothelial cells, pericytes, astrocytes

## Abstract

Tight regulation of positive and negative regulators of angiogenesis is essential, particularly in the eye where their dysregulation can lead to vision loss. Thrombospondin-1 (TSP1) is a matricellular protein that negatively regulates angiogenesis and inflammation in the eye. It aids ocular vascular homeostasis such that its loss contributes to increased retinal vascular density and pathologic ocular neovascularization. Our previous studies demonstrated that mice globally lacking TSP1 expression had increased retinal vascular density, decreased hyperoxia-induced retinal vessel loss, and increased choroidal neovascularization. Here we determined the impact to the ocular vasculature of endothelial cell, pericyte, or astrocyte loss of TSP1 expression. Only lack of TSP1 expression in endothelial cells was sufficient to increase choroidal neovascularization with mice lacking expression in pericytes or astrocytes not demonstrating a significant impact. Although the global TSP1 knockout mice demonstrated increased retinal vascular density, individual cell type loss of TSP1 resulted in decreased retinal endothelial cell numbers before and/or after vascular maturation in a cell type specific fashion. Retinas from mice lacking TSP1 expression in endothelial cells, pericytes or astrocytes were not protected from retinal vessel regression in response to hyperoxia as we previously observed in the global knockout. Thus, modulation of TSP1 expression in individual cell types demonstrates a response that is unique to the role TSP1 plays in that cell type of interest, and their coordinated activity is critical for vision.

## Introduction

The growth of new blood vessels from existing capillaries is a highly regulated process. This is accomplished by opposing action of pro- and anti-angiogenic factors, and their alteration in various pathologies promote the growth of new blood vessels. Thrombospondin-1 (TSP1) was one of the first anti-angiogenic factors whose expression was decreased during transformation and tumor growth (Dameron et al., 1994). Numerous studies have now established changes in TSP1 expression as a key event in the angiogenic switch during tumor growth and metastasis (Sheibani and Frazier, 1999). Furthermore, the angioinhibitory activity of TSP1 is mimicked by a seven amino acid peptide expanding an overlapping region in TSP1, namely the collagen homology and Type I repeats (Tolsma et al., 1993). This peptide has antiangiogenic activity in various angiogenesis models including laser-induced choroidal neovascularization and ocular uveal melanoma tumor growth (Wang et al., 2012a, b).

We showed expression of TSP1 is important in turning off the angiogenic phenotype of endothelial cells in culture (Sheibani and Frazier, 1995). Brain endothelial cells (Bend3.1) that are highly angiogenic and form hemangiomas in mice produce lower amounts of TSP1. Restoration of TSP1 levels is sufficient to establish a normal gene expression profile and suppresses tumor forming ability of Bend3.1 cells. In addition, retinal endothelial cells deficient in TSP1 expression proliferate faster and show enhanced migratory activity, i.e., more angiogenic (Wang et al., 2006). These observations are consistent with previously noted increased vascular density in the retinas from TSP1-deficient mice (Wang et al., 2003). These mice also showed enhanced choroidal vascularization in the laser-induced mouse model (Wang et al., 2012b). Although TSP1 is expressed by other vascular cells including pericytes and astrocytes (Scheef et al., 2005, 2009; Calippe et al., 2017), the impact of TSP1 expression in these cells on ocular vascular homeostasis and neovascularization remains unknown.

Using retinal pericytes and astrocytes prepared from wild type and TSP1 deficient mice we showed TSP1-deficient pericytes exhibit lower proliferation and migration capacity, and fail to respond to promigratory activity of PDGF-BB (Scheef et al., 2009). Retinal astrocytes prepared from TSP1 deficient mice also demonstrate defects in cell adhesive and migratory properties (Scheef et al., 2005). In addition, others have shown expression of TSP1 and TSP2 by astrocytes is essential for synaptogenesis of retinal ganglion cells (RGC) (Christopherson et al., 2005). We showed an increase in expression of TSP2 in TSP1-deficient astrocytes suggesting a potential compensation for some of the TSP1 activity in these cells (Scheef et al., 2005). However, we did not note a significant impact for TSP2 deficiency in retinal vascular development or neovascularization (Fei et al., 2015). Thus, the impact of TSP expression on retinal vascular development and neovascularization is unique to TSP1. How TSP1 expression in pericytes and astrocytes affect retinal vascular development and neovascularization require further evaluation.

Here we describe the generation of a targeted TSP1 transgenic line to investigate the cell specific impact TSP1 expression has on retinal vascular development and neovascularization. We show that TSP1 expression in endothelial cells and mononuclear phagocytes play important roles in ocular vascular homeostasis and ocular neovascularization and inflammation.

## Materials and Methods

### Ethics Statement

The experiments conducted in this study were performed in accordance to the “Association for Research in Vision and Ophthalmology Statement for the Use of Animals in Ophthalmic and Vision Research”. The animal study protocols were approved by the “Institutional Animal Care and Use Committee” of the University of Wisconsin School of Medicine and Public Health. Euthanasia by CO_2_ asphyxiation was performed according to approved protocols.

### Animals

We maintained *Gfap*-Cre [B6.Cg-Tg (*Gfap*-Cre) 73.12Mvs/J; Jackson Laboratory stock number 012886], *Thbs1*^Flox/Flox^ (Yang et al., 2020), Tg(*Pdgfrb*-Cre)*^45*Vli*^* (Cuttler et al., 2011), *Lyz2*-Cre (B6.129P2-*Lyz2tm1(cre)Ifo*/J; Jackson Laboratory stock number 004781), and *Cdh5* (VE-cadherin)-Cre [B6.Cg-Tg(*Cdh5*-cre)7Mlia/J; Jackson Laboratory, Bar Harbor, ME; stock number 006137] mice at the University of Wisconsin vivarium. Genotyping of the Tg(*Pdgfrb*-Cre)*^45*Vli*^* mouse line was done using primers 5′-GCATTTCTGGGGATTGCTTA-3′ and 5′-CCCGGCAAAACAGGTAGTTA-3′, *Gfap*-Cre mice with 5′-TCCATAAAGGCCCTGACATC-3′ and 5′-TGCGAACCTCATCACTCGT-3′, and *Thbs1*^Flox/Flox^ mice with 5′-GGTTTTCCTATTACACTCAGCC-3′ and 5′-GTTCAAAGCCACGCTTGTAAG-3′, and *Lyz2*-Cre with 5′′-CCCAGAAATGCCAGATTACG-3′, 5′-CTTGGGCTGCCA GAATTTCTC-3′, and 5′-TTACAGTCGGCCAGGCTGAC-3′. We bred mice homozygous for the floxed allele (*Thbs1*^*Flox/Flox*^) with Tg(*Pdgfrb*-Cre)*^45*Vli*^*, *Cad5*-Cre or *Gfap*-Cre mice. This generated TSP1^*Flox/+*^ mice that expressed Pdgfrb-Cre, *Cad5*-Cre or *Gfap*-Cre. These mice were then bred and the offspring genotyped as noted above. This strategy allowed us to generate TSP1^Flox/Flox^ mice that also expressed the Cre of interest. These conditional breeding colonies were maintained by breeding *Thbs1*^Flox/Flox^ mice to Cre-expressing *Thbs1*^Flox/Flox^ mice. All progenies were genotyped.

Mice homozygous for the *Thbs1*^Flox/Flox^ allele that express *Pdgfrb*-Cre are referred to as TSP^PC^ (pericyte targeted) mice, those expressing *Gfap*-Cre are referred to as TSP^AC^ (astrocyte targeted) mice, expressing *Cad5*-Cre referred to TSP^EC^ (endothelial cell targeted) and those expression *Lyz2*-Cre referred to as TSP^MP^ (mononuclear phagocyte targeted) mice (Yang et al., 2020). *Thbs1*^Flox/Flox^ mice are referred to as control or wild-type littermates at times. To validate specificity of Cre-mediated excision, we generated mice carrying a conditional Tomato allele and *Pdgfrb*-Cre, *Cad5*- or *Gfap*-Cre. We noted Tomato expression in retinal pericytes/vascular smooth muscle cells (PC), endothelial cells (EC) or astrocytes (AC) (Zaitoun et al., 2015, 2019; Wang et al., 2017; Yang et al., 2020). Characterization of TSP^MP^ mice was recently published (Yang et al., 2020). Male and female mice were used in all the experiments shown below.

### Oxygen-Induced Ischemic Retinopathy

For oxygen-induced ischemic retinopathy (OIR) mouse model, we exposed 7-days-old (P7) pups with dams to an atmosphere of 75 ± 0.5% oxygen for 5 days with the incubator temperature kept at 23 ± 2°C. Oxygen was continuously monitored with a PROOX model 110 oxygen controller (BioSpherix Ltd.; Parish, NY, United States). Retinal wholemounts were prepared from P12 mice as previously described (Wang et al., 2005, 2011; Jamali et al., 2017).

### Visualization of Retinal Vasculature

Eyeballs were enucleated from male and female mice and briefly fixed in 4% paraform-aldehyde (10 min on ice). They were then placed in methanol at −20°C for a minimum of 24 h as we previously described (Wang et al., 2005, 2017; Zaitoun et al., 2015). Retinas that were kept in blocking solution with anti-collagen IV (Millipore, Burlington, MA, United States; AB756P) [diluted 1:250 in blocking solution; PBS containing 20% normal goat serum, 20% fetal calf serum (FCS)] overnight at 4°C were then incubated with an appropriate secondary antibody (Jackson ImmunoResearch Laboratories, West Grove, PA, United States), mounted, examined using fluorescence microscopy and capturing images in digital format using a Zeiss microscope (Carl Zeiss, Chester, VA, United States). In OIR studies the central vessel obliteration area (as percentage of the whole retina area) in digital images was quantified as previously described (Jamali et al., 2017). This was performed in a masked fashion.

### Trypsin-Digested Retinal Vessel Preparation

Eyes were enucleated and fixed in 4% paraformaldehyde for at least 24 h. Next, eyeballs were dissected and whole retina was removed under a dissecting microscope. Retinal trypsin digests were prepared, and PAS stained as we previously described (Wang et al., 2005, 2017). We used nuclear morphology to identify pericytes from endothelial cells. The number of pericytes and endothelial cells on retinal capillaries was determined masked by counting the number of nuclei per field of view under the microscope at a magnification of ×400. Counts were performed on vasculature that corresponds to the middle (mid-zone) of the retina by counting the number of pericytes and endothelial cells in four fields of view from the four quadrants of each retina. Eyes from at least five mice were used for each time point.

### Laser Induced Choroidal Neovascularization

Mice (8-weeks-old male and female) were anesthetized with ketamine hydrochloride (80 mg/kg) and xylazine (10 mg/kg), and Tropicamide (1%) was used to dilate their pupils. The Bruch’s membrane was ruptured by laser photocoagulation. A slit lamp delivery system of an OcuLight GL diode laser (Iridex, Mountain View, CA, United States) was used to locate the 9, 12, and 3 o’clock positions of the posterior pole of each eye for laser photocoagulation (75 μm spot size, 0.1 s duration, 120 mW) using a handheld cover slip as a contact lens to view the retina. After 14 days, the eyes were enucleated, fixed in 4% paraformaldehyde, and washed in PBS. The eyes were sectioned at the equator. This was followed by removal of the retina, vitreous and the anterior half. The rest of the eye was then submerged in blocking buffer (20% normal goat serum and 5% FCS in 1xPBS) for 30–60 min. The choroid/RPE was incubated with anti-ICAM-2 (BD Pharmagen, #553326; 1:500 in 1xPBS with 20% normal goat serum and 20% FCS) at 4°C overnight. The tissue was washed and incubated with the appropriate secondary antibody. The choroid/RPE complex was flatmounted and images were captured in digital format using a Zeiss microscope (Zeiss, Chester, VA, United States). The total area (in μm^2^) of CNV for individual laser burns were measured using Image J software (National Institute of Mental Health, Bethesda, MD, United States^1^).

### RNA Purification and Real Time qPCR Analysis

Retinal total RNA was extracted using mirVana PARIS kit (Invitrogen) and 1 μg total RNA was used for cDNA synthesis with a Sprint RT Complete-Double PrePrimed kit (Clontech, Mountain View, CA, United States). For qPCR, cDNA (1 μl each diluted 1:10) was used and assays were performed in triplicate of three biological replicates using the Mastercycler Realplex (Eppendorf) and SYBR-Green qPCR Premix (Clontech). Amplification parameters were as follows: 95°C for 2 min; 40 cycles of amplification (95°C for 15 s, 60°C for 40 s); dissociation curve step (95°C for 15 s, 60°C for 15 s, 95°C for 15 s). We used *Thbs1* primers 5′-TGGCCAGCGTTGCCA-3′ (forward) and 5′-TCTGCAGCACCCCCTGAA-3′ (reverse) with *RpL13a* 5′-TCTCAAGGT TGTTCG GCTGAA-3′ (forward) and 5′-CCAGAC GCCCCAGGTA-3′ (reverse). Standard curves were generated from known quantities of each target gene with linearized plasmid DNA. We used ten times dilution series for each known target, which we amplified using SYBR-Green qPCR. The linear regression line for DNA (ng) was assessed from relative fluorescent units (RFU) at a threshold fluorescence value (Ct). Gene targets were quantified from cell extracts by comparing the RFU at the Ct to the standard curve and normalized by the simultaneous amplification of RpL13a, a housekeeping gene.

### Statistical Analysis

We evaluated statistical differences between groups with an ANOVA with Tukey’s Multiple Comparison Test using GraphPad Prism version 5.04 for Windows (GraphPad Software, La Jolla, CA, United States). Mean ± standard deviation is shown. We then confirmed the comparison between wild-type and each conditional deletion with Bonferroni’s comparison and student’s unpaired *t*-test (two-tailed). Mean ± standard deviation is shown. *P* < 0.05 was considered significant.

## Results

### Decreased Pericyte and Endothelial Numbers in TSP^PC^ Mice

**FIGURE 1:**
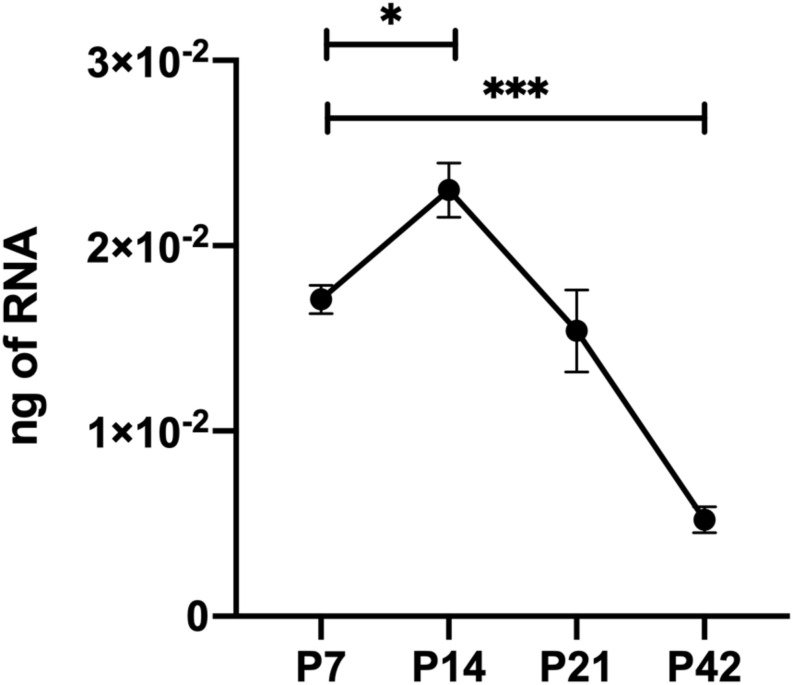
Thrombospondin-1 (TSP1) expression during retinal vascularization. RNA was prepared from retinas of C57BL/6j mice at the noted times during retinal vascular development and maturation. qPCR was utilized to analyze TSP1 expression as noted in section “Materials and Methods”. Please note increased TSP1 expression at P14 with subsequent decline at P42. ^∗^*P* < 0.05, ^∗∗∗^*P* < 0.001.

Thrombospondin-1 is an endogenous inhibitor of angiogenesis that has a well-established role stemming off pathologic ocular neovascularization. TSP1 is expressed in many cell types throughout retinal vascular development but its expression wanes at maturation following remodeling (Figure 1) (Suzuma et al., 1999; Fei et al., 2015). In addition, TSP2 expression in the retina showed a similar pattern of developmentally regulated expression as TSP1 (Fei et al., 2015). Although TSP2 deficiency was associated with reduced expression of TSP1, TSP1 deficiency did not affect the expression pattern and levels of TSP2 in retina during development and OIR (Fei et al., 2015). Our previous research demonstrated that mice lacking TSP1 exhibited increased vascular density mainly due to increased endothelial cell numbers (Wang et al., 2003), a change that it is not mimicked by TSP2 deficiency (Fei et al., 2015). When TSP1-deficient retinal endothelial cells were grown in culture their proangiogenic phenotype remained (Su et al., 2003), however, the same could not be said for TSP1-deficient retinal pericytes or astrocytes (Scheef et al., 2005, 2009). Here we generated *Thbs1*^Flox/Flox^ mice, which also expressed *Cad5-*Cre (TSP^EC^), *Pdgfrb*-Cre (TSP^PC^) or *Gfap*-Cre (TSP^AC^) to address the contribution TSP1 has in individual cell types in the retinal vasculature.

**FIGURE 2:**
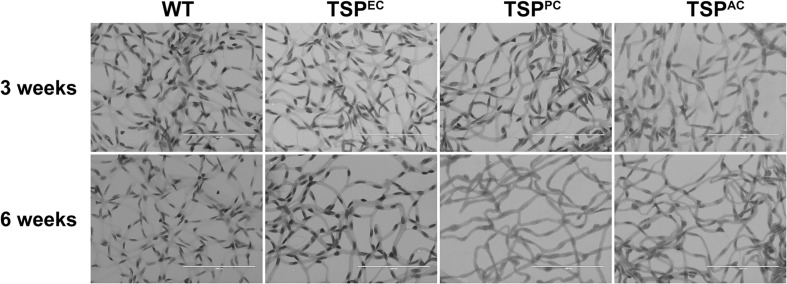
Endothelial cell and pericyte numbers from mice conditionally lacking TSP1. Retinas from 3- and 6-week-old mice were prepared by trypsin digest and HE/PAS staining. Endothelial cell and pericyte numbers were determined per ×400 field of view. Scale bar = 100 μm. Experiments were repeated with eyes from >10 mice with similar results. Table 1 is the quantitative summarization of this data.

The retinal vasculature is laid down by 3 weeks of age with remodeling then occurring until 6 weeks of age (Wang et al., 2003). We did not note any significant differences in postnatal retinal vascularization of global TSP1-deficent mice up to 3-weeks of age compared with wild type mice. However, the retinal vascular density of 6-weeks-old TSP1-deficient mice was significantly greater than wild type mice (Wang et al., 2003). Here we assessed the numbers of endothelial cells and pericytes using trypsin digest preparations from wild-type, TSP^EC^, TSP^PC^ and TSP^AC^ mice at 3 and 6 weeks of age. In Figure 2, nuclei that appear large and oval within the vessel wall and are weakly stained belong to endothelial cells. In contrast, nuclei that are small round and darkly stained protruding laterally from the vessel wall are counted as pericytes. Table 1 is a quantified assessment of these trypsin digest preparations. At 3 weeks, mice lacking TSP1 expression in endothelial cells demonstrated increased numbers of pericytes, but similar endothelial cell levels compared to controls. Following retinal vascular remodeling at 6 weeks, the numbers of endothelial cells in mice lacking endothelial cell TSP1 expression significantly decreased compared to control mice.

**TABLE 1:** Retinal vascular cell numbers.

	Age	WT	TSP^EC^	TSP^PC^	TSP^AC^
Pericytes (PC)	3 weeks	35.75 ± 1.22	43.75 ± 1.94**	31.92 ± 1.54	32.04 ± 1.28*
Endothelial cell (EC)	3 weeks	146.85 ± 5.96	140.75 ± 5.32	115.17 ± 3.30****	119.79 ± 3.14****
Pericytes (PC)	6 weeks	38.17 ± 1.50	41.88 ± 1.64	26.58 ± 0.66*****	39.67 ± 1.2
Endothelial cell (EC)	6 weeks	114.46 ± 3.34	102.92 ± 3.89*	102.63 ± 4.48*	112.29 ± 3.37

Number of cells per high power field at (×400). The P values were calculated by comparing samples from WT to TSP1^EC^, TSP1^PC^ or TSP1^AC^ mice at the ages noted: *P < 0.05; **P < 0.01; ****P < 0.0001; and *****P < 0.00001.

**FIGURE 3:**
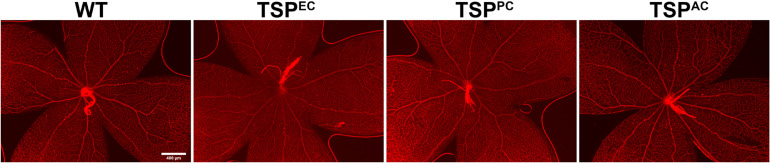
Supercritical retinal vascular layer. Retinal wholemount preparation from P7 mice were stained with anti-collagen IV. Eyes from at least five mice were stained. Please note no gross abnormalities were noted. Scale bar = 400 μm.

TSP1 expression in pericytes influences their proliferation and migration (Scheef et al., 2009). In mice lacking pericyte TSP1 expression, we observed decreased numbers of endothelial cells both at 3 and 6 weeks of age as well as decreased pericyte numbers at 6 weeks of age. In astrocytes TSP1 expression is important for synaptogenesis of RGC (Christopherson et al., 2005). Here we show that mice lacking TSP1 expression in astrocytes demonstrated decreased numbers of endothelial cells compared to controls. These levels did not decrease further with remodeling. These changes did not equate into gross abnormalities in the superficial layer in cell specific absence of TSP1 expression (Figure 3) as we previously observed in mice globally lacking TSP1 (Wang et al., 2003). Thus, when TSP1 is selectively deleted in a single vascular cell-type we observed a much different impact on retinal vascular endothelial and pericyte numbers compared to its complete lack in the organ.

### Lack of TSP1 in Endothelial Cells, Pericytes or Astrocytes Does Not Impact Vessel Obliteration During OIR

**FIGURE 4:**
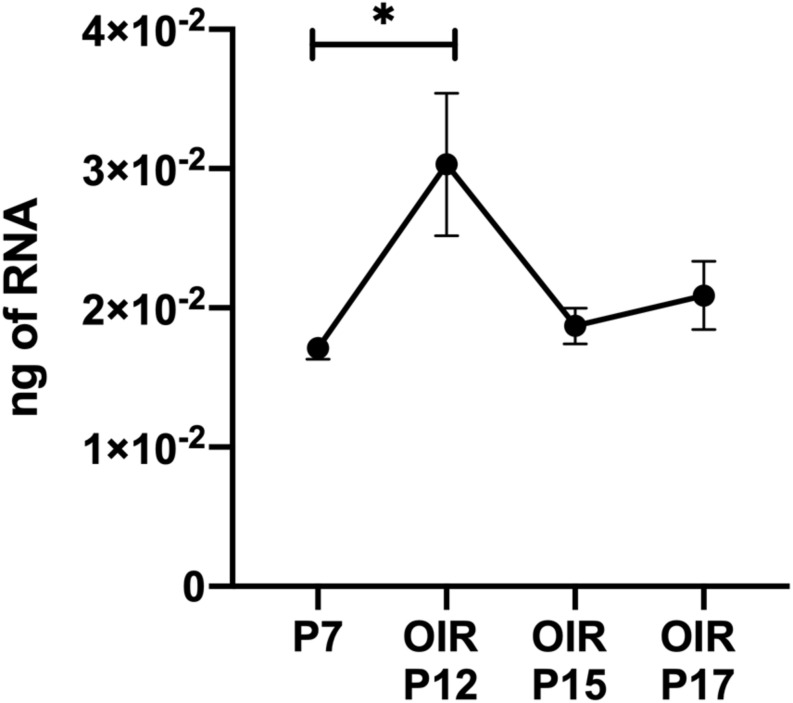
Thrombospondin-1 expression increases during hyperoxia. At P7, C57BL/6j mice were subjected to oxygen-induced ischemic retinopathy (OIR). RNA was extracted from retinas at the noted times and TSP1 was analyzed by qPCR as noted in materials and methods. Please note TSP1 expression increased with hyperoxia (P12). ^∗^*P* < 0.05.

**FIGURE 5:**
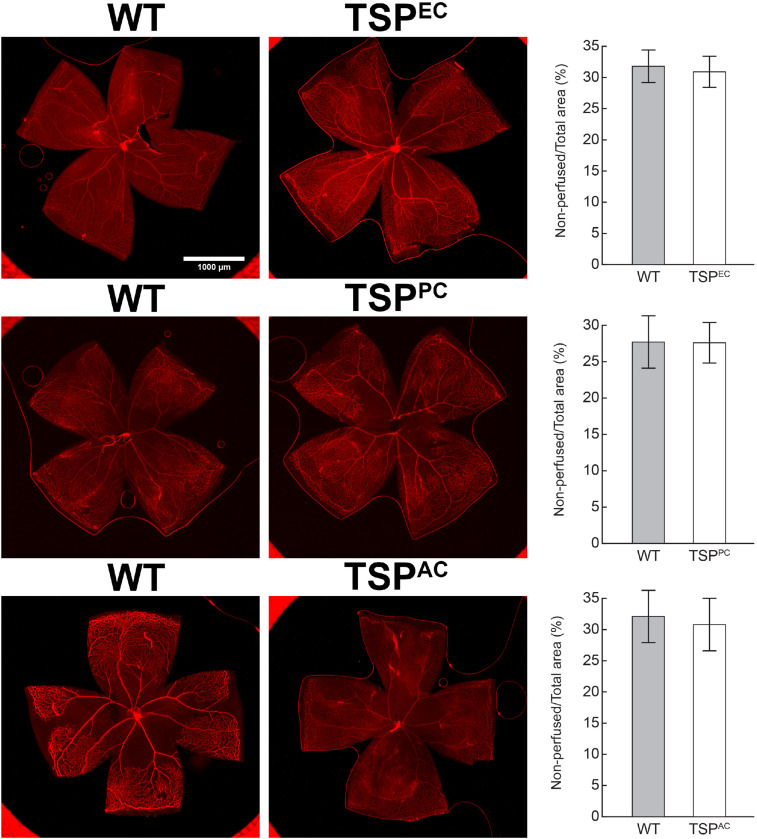
Lack of TSP1 expression in endothelial cells, pericytes or astrocytes did not attenuate hyperoxia-driven vessel obliteration. Vessel obliteration P12 mice exposed to a cycle of hyperoxia beginning at P7 was assessed. Retinas were wholemount stained with anti-collagen IV to visualize the vasculature. The area of vessel obliteration relative to the whole retina was quantitated (*P* > 0.05). Scale bar = 1,000 μm. Experiments were repeated at least three times with 5 mice per group with similar results.

Thrombospondin-1 increases endothelial cell apoptosis particularly under hyperoxic conditions (Wang et al., 2003). This is consistent with augmented TSP1 expression during hyperoxia at P12 and its subsequent equilibration upon return to room air (Figure 4). We previously reported no differences in the expression of TSP1 levels in retinas prepared from P15 mice reared in room air or subjected to OIR (Wang et al., 2003). These observations are also consistent with our previous studies demonstrating that the retinal vasculature was protected from hyperoxia mediated vessel obliteration in TSP1-deficient mice during OIR (Wang et al., 2003). Here we determined whether lack of TSP1 expression in a specific vascular cell type namely endothelial cells, pericytes or astrocytes prevented hyperoxia mediated retinal vessel loss (Figure 5). Mice at P7 were exposed to hyperoxia (75%) for 5 days. The area of hyperoxia vessel loss was assessed at P12 OIR in mice lacking TSP1 expression in various vascular cells. Unlike the global knockout, mice lacking TSP1 expression in endothelial cells, pericytes or astrocytes demonstrated similar levels of hyperoxia vessel loss to control littermates. Thus, to enhance vessel survival during hyperoxia global loss of TSP1 is essential.

### Increased CNV in Mice Lacking Endothelial TSP1 Expression

**FIGURE 6:**
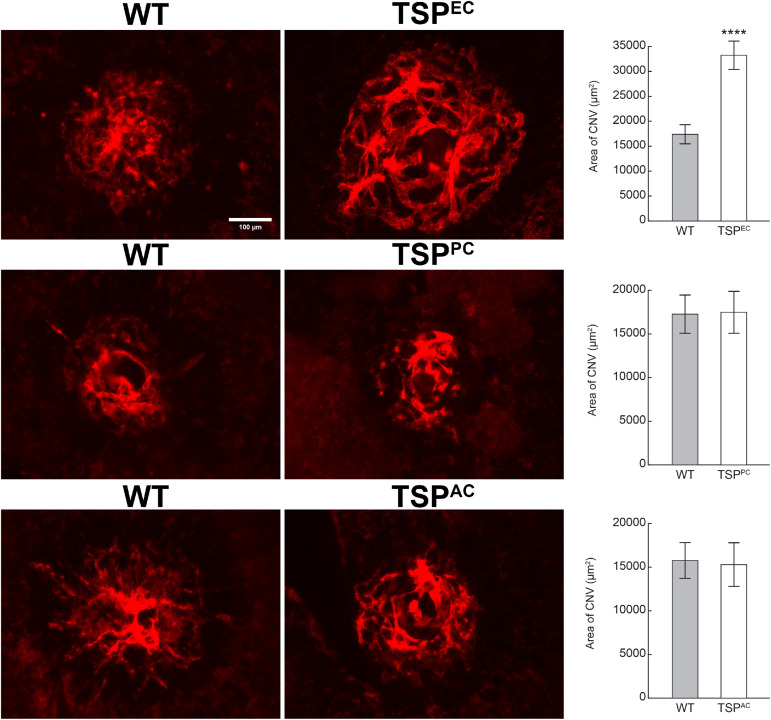
Increased choroidal neovascularization in mice lacking TSP1 expression in endothelial cells. Choroidal neovascularization was induced in 6-weeks old male and female wild type (WT) and TSP1 conditional mice by laser photocoagulation-induced rupture of Bruch’s membrane. After 14 days, the eyes were sectioned at the equator, and the anterior half/vitreous/retina removed. The remaining eye tissue was stained with anti-ICAM-2 and the area of neovascularization quantitated (****P < 0.0001). Scale bar = 100 μm.

**FIGURE 7:**
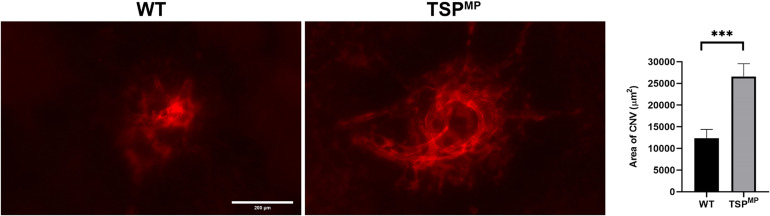
Lack of TSP1 expression in mononuclear phagocytes increases choroidal neovascularization. 6-weeks old male and female *Thbs1*^wt/wt^ Lyz2-Cre and *Thbs1*^Flox/Flox^ Lyz2-Cre mice were subjected to laser photocoagulation-induced rupture of Bruch’s membrane. At 14 days, the eyes were stained with anti-ICAM-2 and the area of neovascularization quantitated (^∗∗∗^*P* < 0.001). Scale bar = 200 μm.

Our previous studies demonstrated that TSP1 restrains choroidal neovascularization (Wang et al., 2012b). Here we assessed whether lack of TSP1 expression in specific vascular cells could increase CNV, as was noted in in global TSP1-deficient mice. Mice underwent laser photocoagulation and 2 weeks later the choroid/RPE complex was stained with anti-ICAM2. Figure 6 demonstrates increased CNV in mice lacking endothelial TSP1 expression while mice lacking pericyte or astrocyte TSP1 expression demonstrated similar levels of CNV as controls. These data are consistent with our studies showing endothelial cells lacking TSP1 demonstrate a proangiogenic phenotype (Su et al., 2003; Wang et al., 2006). Since TSP1 expression in mononuclear phagocytes is essential for their clearance at site of laser induced lesions (Wang et al., 2012b; Calippe et al., 2017), we next asked whether loss of TSP1 expression in mononuclear phagocytes impacts CNV. Our previous studies indicated that lack of TSP1 expression resulted in increased F4/80 positive mononuclear phagocytes at the sites of CNV lesions (Wang et al., 2012b). Here, we show that lack of TSP1 expression in mononuclear phagocytes also resulted in increased CNV as noted in TSP1 global knockout mice (Figure 7). Thus, TSP1 expression in mononuclear phagocytes also impacts the level of CNV through modulation of inflammatory cell activity.

## Discussion

The ability of TSP1 to inhibit angiogenesis has been purported to lay squarely on its influence on endothelial cell proliferation and migration *in vitro* and angiogenesis *in vivo* (Tolsma et al., 1993; Jimenez et al., 2000, 2001) even though TSP1 is expressed in all vascular cell types. During ocular vascular development TSP1 is an active participant. Its early enhanced expression attenuates postnatal retinal vascularization (Wu et al., 2006) while its expression is needed in later stages for remodeling and maturation (Wang et al., 2003). Its expression also acts to prevent aberrant vessel growth into areas that would not be amenable to vascularization (Sheibani et al., 2000). Thus, it is not surprising that the impact TSP1 has in squelching pathologic neovascularization and inflammation is well documented (Masli et al., 2014). Unfortunately, the unique role TSP1 plays in specific retinal vascular cells in the context of the eye is less defined.

In the studies presented here we asked whether targeted deletion of TSP1 expression in endothelial cells, pericytes or astrocytes has a similar impact as its global loss of expression. During normal retinal vascular development endothelial cells are pruned during maturation to remove superfluous vasculature (Fruttiger, 2007). In mice globally lacking TSP1 we previously observed that this pruning did not occur leaving endothelial cell numbers at 3 and 6 weeks similar in TSP1-deficient mice (Wang et al., 2003). Here we show that retinal vascular remodeling still occurs in TSP1^EC^ mice. In contrast in TSP^PC^ and TSP^AC^ mice, we observed lower numbers of endothelial cells at 3 weeks compared to controls which did not decrease further at 6 weeks. Lack of remodeling in these mice was most likely unwarranted due to the low initial endothelial cell numbers. These results suggest an important role for TSP1 expression in pericytes and astrocytes during active phase of retinal vascularization. Thus, the reliance each retinal vascular cell type has on each other during retinal vascularization becomes evident when one peels away the intricate layers by conditionally deleting TSP1 in a specific cell type.

Retinal pericytes lacking TSP1 expression demonstrate decreased proliferation, adhesion, and migration *in vitro* (Scheef et al., 2009). These altered pericyte characteristics are consistent with the decreased numbers of vascular cells observed in TSP^PC^ mice. Astrocytes set the scaffolding for the nascent vasculature formation an activity coordinated by RGC (O’Sullivan et al., 2017). Recent studies have shown that expression of TSPs by astrocytes play key roles in proper synaptogenesis of RGC (Christopherson et al., 2005). Thus, RGC presence is essential for proper vascularization of retina and likely, the proper function of astrocytes. TSP1-deficient astrocytes in culture exhibited increased adhesion and increased TSP2 expression, perhaps compensating for reduced TSP1 levels (Scheef et al., 2005). We previously showed lack of TSP2 was associated with reduced levels of TSP1 expression in the retina with minimal effects on postnatal retinal vascular development and neovascularization during OIR (Fei et al., 2015). In contrast, lack of TSP1 minimally affected TSP2 expression in the retina at various postnatal times and during OIR *in vivo* (Fei et al., 2015). Thus, *in vivo* global deficiency of TSP1 in the retina is not compensated by increased expression of TSP2, which contrasts with our observation in retinal astrocytes in culture. These results suggest that the absence of TSP1 may have adverse effects on astrocyte function and their interaction with RGC *in vivo*, which contribute to the decreased numbers of endothelial cells in the TSP^AC^ mice. Thus, modulating TSP1 expression in a single retinal vascular cell type *in vivo* has a different impact than its global deficiency further exemplifying the complexity of the retinal vascular system and the importance of cell autonomous functions.

The developing retinal vasculature displays an inherent sensitivity to changes in oxygen tension (Chen and Smith, 2007). This results in retinopathy of prematurity in preterm infants. This malady is mimicked in the murine oxygen induced ischemic retinopathy model (Smith et al., 1994). Our previous studies demonstrated that global lack of TSP1 diminished hyperoxia-induced retinal vessel loss (Wang et al., 2003). Here we showed that loss of TSP1 expression in endothelial cells, pericytes or astrocytes was not sufficient to curtail hyperoxia-induced retinal vessel loss. These results further support the notion that other neuronal and glial cells may contribute to impact of globally TSP1 deficient mice, which requires further investigation.

Thrombospondin-1 ability to restrain pathologic neovascularization particularly evident in the eye. Consistent with its decreased expression during solid tumor progression (Lawler and Lawler, 2012) is its reduced expression in a preclinical mouse model of uveal melanoma (Wang et al., 2012a). The decrease in TSP1 expression in *Tyr*-tag mouse model of uveal melanoma correlated with increased vascular density and tumor size, which was suppressed upon overexpression of TSP1 in these mice (Wang et al., 2012a). Furthermore, administration of TSP1 mimetic peptide, ABT-898 was also sufficient to suppress tumor growth. Thus, decreased production of TSP1 makes a significant contribution to the development and progression of uveal melanoma, and its restoration could suppress tumor growth. Thus, appropriate expression of TSP1 in the eye is not only essential to keep the integrity of retinal neurovasculature in check but also limits tumor mediated ocular angiogenesis.

The retinal vasculature is significantly different from that of choroid, but the identity of these differences and the molecular mechanisms involved remain largely unknown. The endothelial cells of the choroidal vasculature, unlike the endothelial cells of the retinal vasculature, are fenestrated (Lutty et al., 2010). This is in agreement with high flow rate associated with the choroidal vasculature for meeting the metabolic needs of the highly active photoreceptor cells. Our studies demonstrated that the choroidal endothelial cells prepared from TSP1-deficient mice exhibit a very different phenotype compared to the endothelial cells from the retina of these mice. TSP1-deficient choroidal endothelial cells were less migratory and proliferative compared to the wild-type choroidal endothelial cells (Fei et al., 2014). The underlying mechanisms for these difference in response to TSP1 expression remains unknown and deserves further investigation.

Thrombospondin-1 also exhibits a well characterized anti-inflammatory activity in the eye, and thus, may also impact ocular neovascularization, especially in the choroid since inflammation plays a major role in choroidal neovascularization and exudative AMD (Hollyfield et al., 2008; Masli et al., 2014). Consistent with this notion, we demonstrated that the TSP1-deficient mice exhibit enhanced choroidal neovascularization (Wang et al., 2012b). This was correlated with increased accumulation of inflammatory mononuclear phagocytes at the lesion sites. A recent study demonstrated that expression of TSP1 is essential for the clearance of the mononuclear phagocytes in the CNV lesions (Calippe et al., 2017). Thus, in global TSP1-deficient mice the delayed clearance of mononuclear phagocytes could contribute to the enhanced choroidal neovascularization. We showed the TSP1 mimetic peptide ABT-898 was efficacious in mitigating choroidal neovascularization in both wild type and TSP1-deficient mice (Wang et al., 2012b). We recently generated a line of mice which lack TSP1 expression in myeloid linage cells using *Lyz2*-Cre mice (Yang et al., 2020). When these mice were subjected to laser induced choroidal neovascularization, we observed a similar enhanced neovascularization. Thus, expression of TSP1 in mononuclear phagocytes could also play a key role in maintaining choroidal vascular homeostasis, whose alterations during aging may contribute to the development and progression of exudative AMD.

## Data Availability Statement

The original contributions presented in the study are included in the article/supplementary material, further inquiries can be directed to the corresponding author.

## Ethics Statement

The animal study was reviewed and approved by Institutional Animal Care and Use Committee of University Wisconsin School of Medicine and Public Health. Written informed consent was obtained from the owners for the participation of their animals in this study.

## Author Contributions

CS was responsible for experimental design, conducting experiments, data analysis and interpretation, and writing and editing the manuscript. SW, SD, and ZG were responsible for experiments and data collection. BL helped with study design, protocol implementation, and editing manuscript. NS and CS were responsible for study design, protocol development, data analysis and interpretation, writing and editing the manuscript, and for the final approval of the article. The final manuscript was read and approved by all authors.

## Conflict of Interest

The authors declare that the research was conducted in the absence of any commercial or financial relationships that could be construed as a potential conflict of interest.

## Footnotes

1 http://rsb.info.nih.gov/ij/
